# Metagenome Sequences of Sediment from a Recovering Industrialized Appalachian River in West Virginia

**DOI:** 10.1128/genomeA.00350-18

**Published:** 2018-05-03

**Authors:** David H. Huber, Ifeoma R. Ugwuanyi, Sridhar A. Malkaram, Natalia A. Montenegro-Garcia, Vadesse Lhilhi Noundou, Jesus E. Chavarria-Palma

**Affiliations:** a Department of Biology, West Virginia State University, Institute, West Virginia, USA; b Gus R. Douglass Institute, West Virginia State University, Institute, West Virginia, USA

## Abstract

We sequenced two metagenomes from upper sediment layers (0 to 5 and 6 to 10 cm) from the Kanawha River, West Virginia. The watershed includes inputs from the forested Appalachian Mountains, surface coal mining, municipal residues, and extensive chemical manufacturing. The dominant bacterial phyla were Proteobacteria, Bacteriodetes, Firmicutes, Actinobacteria, and Chloroflexi. Xenobiotic degradation pathways were present.

## GENOME ANNOUNCEMENT

Rivers and watersheds provide essential resources for municipal, industrial, and agricultural processes. However, heavy use often degrades water quality and ecosystem services. The maintenance and resilience of biodegradation and bioremediation functions in watersheds is linked to microbial diversity of the sediment. River sediment microbiomes are among the most biodiverse microbiomes known, yet they are still poorly sampled (1–3). The Kanawha River serves the industrial heart of West Virginia, running through the Charleston metropolitan area and the region known as “Chemical Valley,” which has been highly industrialized with chemical manufacturing since the early 20th century. The Kanawha-New River Basin watershed encompasses more than 12,000 square miles of the Appalachian Mountains, including considerable forests and surface coal mining (4). Downstream from Charleston, the Kanawha River was considered to be one of the most polluted rivers in the United States during the 1950s and 1960s, and it routinely became anoxic in the summers prior to 1983 (5). However, river quality has improved considerably since then. For example, our measurements using a Manta 2 automated sonde (Eureka) found normal levels of oxygen at 12.9 ± 1.1 mg · liter^−1^ from hourly sampling during a recent 4-month period. The purpose of our research is to understand how local and regional stressors affect microbial riverine ecosystem processes.

We collected river sediment samples from the downstream end of Blaine Island (38°22′21.8″ N, 81°41′48.3″ W) on 1 July 2015. Blaine Island is a completely industrialized island in the center of the Kanawha River in South Charleston, WV. A 6.5-cm diameter core was split into two layers (0 to 5 and 6 to 10 cm) for sequencing. Sediment samples were transported on ice and stored in the dark at 4°C for 1 day. From 10 g of sediment for each layer, DNA was extracted with the DNeasy PowerMax soil kit (Qiagen) and stored at −80°C. Shotgun metagenome sequencing was performed by the Oklahoma Medical Research Foundation with the Illumina HiSeq 2500 platform using rapid mode, V2 chemistry, and 2 × 150-bp paired ends. Quality checking was done with FastQC (https://www.bioinformatics.babraham.ac.uk/projects/fastqc/). Adapter trimming and quality filtering were done with Cutadapt (6) and Trim Galore! (7). The total numbers of remaining sequences were 29,439,391 (14,425,165 paired ends) for the top layer (sample RST) and 30,863,794 (15,323,478 paired ends) for the bottom layer (sample RSB). The metagenome from the upper layer contained 2.32 Gb, and that from the bottom layer contained 2.42 Gb. Taxonomic and functional profiling with the Metagenomics Rapid Annotations using Subsystems Technology (MG-RAST) server (8) showed that the dominant phyla in both layers were Proteobacteria (51%), Bacteroidetes (9%), Firmicutes (7%), Actinobacteria (5.5%), Chloroflexi (3.7%), Acidobacteria (3.4%), and Verrucomicrobia (3 to 4%). The most abundant genera were Geobacter (2%), “*Candidatus* Solibacter” (1.8 to 2%), Anaeromyxobacter (2.0%), and Burkholderia (1.8 to 2%). Functional characterization with the Kyoto Encyclopedia of Genes and Genomes (KEGG) database showed the presence of genes associated with bioremediation pathways, including those for the breakdown of polycyclic aromatic hydrocarbons, toluene, xylene, atrazine, and other industrial chemicals.

### Accession number(s).

The metagenome sequences acquired for this project have been deposited in the NCBI database under the Sequence Read Archive accession numbers SRX3771534 and SRX3771535.
